# Different multiparametric MRI-based radiomics models for differentiating stage IA endometrial cancer from benign endometrial lesions: A multicenter study

**DOI:** 10.3389/fonc.2022.939930

**Published:** 2022-08-05

**Authors:** Qiu Bi, Yaoxin Wang, Yuchen Deng, Yang Liu, Yuanrui Pan, Yang Song, Yunzhu Wu, Kunhua Wu

**Affiliations:** ^1^ Department of MRI, The First People’s Hospital of Yunnan Province, The Affiliated Hospital of Kunming University of Science and Technology, Kunming, China; ^2^ Department of Radiology, The First Affiliated Hospital of Chongqing Medical University, Chongqing, China; ^3^ State Key Laboratory of Ultrasound in Medicine and Engineering, College of Biomedical Engineering, Chongqing Medical University, Chongqing, China; ^4^ MR Scientific Marketing, Siemens Healthineers, Shanghai, China

**Keywords:** endometrial cancer, magnetic resonance imaging, radiomics, nomogram, benign endometrial lesions

## Abstract

**Purpose:**

The aim of this study was to evaluate the value of different multiparametric MRI-based radiomics models in differentiating stage IA endometrial cancer (EC) from benign endometrial lesions.

**Methods:**

The data of patients with endometrial lesions from two centers were collected. The radiomics features were extracted from T2-weighted imaging (T2WI), diffusion-weighted imaging (DWI), apparent diffusion coefficient (ADC) map, and late contrast-enhanced T1-weighted imaging (LCE-T1WI). After data dimension reduction and feature selection, nine machine learning algorithms were conducted to determine which was the optimal radiomics model for differential diagnosis. The univariate analyses and logistic regression (LR) were performed to reduce valueless clinical parameters and to develop the clinical model. A nomogram using the radscores combined with clinical parameters was developed. Two integrated models were obtained respectively by the ensemble strategy and stacking algorithm based on the clinical model and optimal radiomics model. The area under the curve (AUC), clinical decisive curve (CDC), net reclassification index (NRI), and integrated discrimination index (IDI) were used to evaluate the performance and clinical benefits of the models.

**Results:**

A total of 371 patients were incorporated. The LR model was the optimal radiomics model with the highest average AUC (0.854) and accuracy (0.802) in the internal and external validation groups (AUC = 0.910 and 0.798, respectively), and outperformed the clinical model (AUC = 0.739 and 0.592, respectively) or the radiologist (AUC = 0.768 and 0.628, respectively). The nomogram (AUC = 0.917 and 0.802, respectively) achieved better discrimination performance than the optimal radiomics model in two validation groups. The stacking model (AUC = 0.915) and ensemble model (AUC = 0.918) had a similar performance compared with the nomogram in the internal validation group, whereas the AUCs of the stacking model (AUC = 0.792) and ensemble model (AUC = 0.794) were lower than those of the nomogram and radiomics model in the external validation group. According to the CDC, NRI, and IDI, the optimal radiomics model, nomogram, stacking model, and ensemble model achieved good net benefits.

**Conclusions:**

Multiparametric MRI-based radiomics models can non-invasively differentiate stage IA EC from benign endometrial lesions, and LR is the best machine learning algorithm. The nomogram presents excellent and stable diagnostic efficiency.

## Introduction

Endometrial cancer (EC) and endometrial hyperplasia and polyps are the most common malignant and benign uterine endometrial cavity lesions, respectively (1). In order to avoid insufficient curing or excessive treatment and to protect the patient’s fertility, it is necessary to accurately identify benign and malignant endometrial lesions before operation. Although endometrial samplings such as dilatation and curettage, endometrial cytology, and biopsy can preoperatively identify some endometrial lesions (2), they do not always provide a definitive diagnosis. Because these procedures are often performed in a blind manner, they may be subject to sampling error and cannot properly diagnose focal endometrial lesions (3). Furthermore, they are difficult to perform in patients with pelvic organ prolapse and vaginal or cervical stenosis (4). In addition, endometrial sampling procedures are invasive with some complications including pain, discomfort, and bleeding. Hence, it is important to find a noninvasive method to distinguish benign and malignant uterine lesions.

Magnetic resonance imaging (MRI) with excellent soft tissue contrast resolution plays an important role in the preoperative diagnosis and staging of EC in situations where it is difficult to obtain histologic samples, and is more sensitive than transvaginal sonography for diagnosing endometrial lesions (1, 5). Multiparametric MRI, including T2-weighted imaging (T2WI), contrast-enhanced MRI (CE-MRI), diffusion-weighted imaging (DWI), and apparent diffusion coefficient (ADC) are increasingly being applied for diagnosing various endometrial lesions (1). However, conventional imaging evaluation of the uterine cavity lesions may present many challenges to the radiologist. The endometrium structure is susceptible to age, menopausal status, menstrual cycle, and hormonal replacement therapy (6). There are a variety of appearances and overlapping imaging features of early-stage EC and benign mimickers (7). Moreover, the experience of the radiologist usually contributes to high interobserver variation. All of these factors lead to inaccurate diagnoses.

Radiomics is an emerging field of application of artificial intelligence in medical imaging by extracting high-throughput quantitative image features and is a problem-solving tool when there is a dilemma in conventional imaging diagnosis (8). Recently, MRI-based radiomics has been gradually applied in the evaluation of EC including risk stratification (9–11), lymph node metastasis (12–14), myometrial invasion (15–17), prognosis and recurrence (18–20), and histological characteristics (21–23). Chen et al. (24) had confirmed that MRI-based radiomics was a valuable tool for distinguishing EC from benign mimics. However, they included stage IB to IV ECs that were easily distinguishable from benign uterine lesions, and only one machine learning algorithm model was studied. Therefore, this study aims to compare the performance of various multiparametric MRI-based machine learning radiomics models in differentiating stage IA EC from benign endometrial lesions, and further assess the potential utility of diverse integrated models utilizing clinical parameters and radiomics features.

## Materials and methods

### Study population

Ethical approval was obtained for this retrospective study, and written informed consent was waived. Between January 2017 and June 2021, consecutive patients with endometrial lesions from center A and center B were collected. Inclusion criteria were as follows: (1) patients with stage IA EC, endometrial hyperplasia, or endometrial polyps confirmed by histopathology; (2) underwent MR examination including T2WI, DWI, and dynamic contrast-enhanced (DCE-MRI) within 2 weeks prior to treatment; and (3) complete clinical data. Exclusion criteria were as follows: (1) MRI quality did not meet the requirement of analysis; (2) received treatment before the MR examination; (3) the maximum diameter of the lesion was less than 1 cm; and (4) patients with other pelvic diseases. Patients from center A were randomly allocated into the training group and the internal validation group at a ratio of 3:1. All patients from center B served as the external validation group. Clinical and histological characteristics of all patients, including histological subtypes, age, menopause, clinical manifestation, metabolic syndrome, body mass index (BMI), actual treatment options, and CA125 and CA199 level, and immunohistochemical findings such as estrogen receptor (ER), progesterone receptor (PR), P53, and Ki-67 were collected.

### Imaging acquisition and lesion segmentation

All MR examinations were performed using 1.5/3.0-T scanners (GE Signa HDXt, Siemens Prisma, and Siemens Aera) with eight-channel phased-array abdominal coils. Each patient underwent preoperative MR scanning using the standard protocol. In the study, uterus-axial T2WI, DWI (b-value = 1,000 s/mm^2^), ADC map, and late contrast-enhanced T1-weighted imaging (LCE-T1WI) were acquired for lesion segmentation. Parameter details are shown in **Table 1**. Some parameters would be adjusted according to the individual differences of patients. The ADC map was automatically reconstructed and generated after scanning DWI by the Siemens MRI scanners, or manually reconstructed on the Functool Software (ADW 4.7 Workstation) by the GE MRI scanner. CE-T1WI was performed immediately after administering a standard dose (0.1 mmol/kg) of gadopentetate dimeglumine (Magnevist; Bayer Healthcare Pharmaceuticals, Germany) at approximately 2 ml/s *via* the elbow vein. Uterus-axial LCE-T1WI was obtained at 240 s into the examination after the contrast agent injection.

**Table 1:** The parameter details of primary sequences.

		Repetition time (ms)	Echo time (ms)	Field of view (mm^2^)	Matrix	Slice thickness (mm)	Slice gap (mm)
Siemens Prisma 3.0 T	T2WI	3,200	90	200 × 200	320 × 320	3	3.6
	DWI	6,300	75	250 × 134	72 × 134	3	3.6
	LCE-T1WI	2.9	1.19	220 × 200	288 × 262	3	0
GE Signa HDXt 3.0T	T2WI	3,500	104	200 × 200	240 × 240	3	1.5
	DWI	4,250	70	200 × 200	240 × 240	3	1
	LCE-T1WI	3.26	1.6	240 × 240	350 × 350	3	1.5
Siemens Aera 1.5 T	T2WI	3,900	90	320 × 320	512 × 512	3	1.5
	DWI	5,600	90	200 × 200	256 × 256	4	1
	LCE-T1WI	3.41	1.3	240 × 240	320 × 320	2	1.5

T2WI, T2-weighted imaging; DWI, diffusion-weighted imaging; LCE-T1WI, late contrast-enhanced T1-weighted imaging.

The original MR images of uterus-axial T2WI, DWI, ADC map, and LCE-T1WI in Digital Imaging and Communications in Medicine (DICOM) format were loaded into 3D Slicer 4.11.0 software (https://www.slicer.org/). Region of interest (ROI) of the lesion was manually delineated layer by layer to form three-dimensional (3D) volume of interest (VOI) by two radiologists (reader 1 and reader 2, with 3 years and 7 years of experience in pelvic MRI, respectively), with unknown clinical information and pathological diagnosis. Reader 1 delineated the boundary of all lesions on uterus-axial T2WI, DWI, and LCE-T1WI, respectively. After 2 months, reader 1 and reader 2 randomly selected the same 50 patients to outline. Care was taken to avoid including endometrial cavity fluid and hematocele and nearby normal myometrium, but necrotic, bleeding, and cystic areas inside the tumor can be included.

### Feature extraction and selection

The open-source Python package Pyradiomics (https://pypi.org/project/pyradiomics/) was used to extract radiomics features from the VOI of each patient at the 3D Slicer platform. To obtain isotropic voxels, the VOIs were resampled to 3 × 3 × 3 mm, then cubic spline interpolation was performed. In order to reduce the imaging differences among different MRI scanners, image normalization was performed so that all gray-level values in the images were distributed in the range of 0–600. A fixed bin width of 1 was selected to ensure better comparability of MRI gray values as suggested in a previous study (12). Before feature extraction, several built-in filters such as gradient, exponent, logarithm, square, square root, wavelet, and Laplacian of Gaussian (LOG) filters were applied on the normalized MR images, and derived images were achieved. The extracted features were divided into the following categories (25): first-order features, two-dimensional features, gray-level co-occurrence matrix (GLCM), gray-level dependence matrix (GLDM), gray-level size-zone matrix (GLSZM), gray-level run-length matrix (GLRLM), and neighboring gray tone difference matrix (NGTDM). A total of 1,781 radiomics features were extracted from each MRI modality, resulting in 7,124 radiomics features for each patient in total. All the above features were standardized by the *Z* score.

The datasets of the patients with stage IA EC and benign endometrial lesions were balanced by using the synthetic minority oversampling technique in the training group. To ensure repeatability and avoid the subjective difference in lesion segmentation, the intraclass correlation coefficient (ICC) of each feature was calculated. Only features with ICC values ≥ 0.75 between observers and within observers were retained. Pearson correlation coefficients were calculated for identifying redundant features. If the correlation coefficient of two features was ≥ 0.9, the feature with the largest mean absolute correlation was deleted. Whereafter, least absolute shrinkage and selection operator (LASSO) was used to select the most representative features and 10-fold cross-validation was performed (26).

### Model building

On the construction of the clinical model, firstly, univariate analysis was conducted to compare the clinical characteristics of benign and malignant endometrial lesions in the training group, and find out the clinical parameters with statistically significant difference. Secondly, the individual predictors of stage IA EC were chosen according to the univariate logistic regression (LR) analysis. Finally, the clinical model was constructed based on the multivariate LR, and the efficient clinical predictive parameters were selected.

Different radiomics models were developed and tested respectively to predict stage IA endometrial cancer based on the following nine machine learning classification algorithms: LR, support vector machine (SVM), stochastic gradient descent (SGD), K nearest neighbor (KNN), decision tree (DT), random forest (RF), extremely randomized trees (ET), eXtreme Gradient Boosting (XGBoost), and Light Gradient Boosting Machine (LightGBM). A fivefold cross-validation strategy was applied to tune and optimize the model parameter, and assess the performance of the models. Referring to a recently published study (27), the machine learning algorithm with the highest average area under the receiver operating characteristic (ROC) curve (AUC) of the internal and external validation group was used to construct the optimal radiomics model. Then, the radiomics score (radscore) was calculated. A nomogram based on the multivariate LR analysis was developed by using the combination of clinical predictive parameters and radscore in the training group.

The stacking model is an integrated learning technology, which can combine the predictions of learned classifiers in order to create prediction of new instances to improve overall performance (28). In the study, a two-tier stacking model was conducted; the first tier was the above clinical model and the optimal radiomics model, and the second tier used the output of the first tier as the input of the multivariate LR. The ensemble algorithm is developed using superlearner (29), and belongs to an integrated strategy. According to the accuracy weight, the predictions obtained from the foregoing clinical model and radiomics model were calculated by the weighted average method and the new output as the final results.

**Figure 1:**
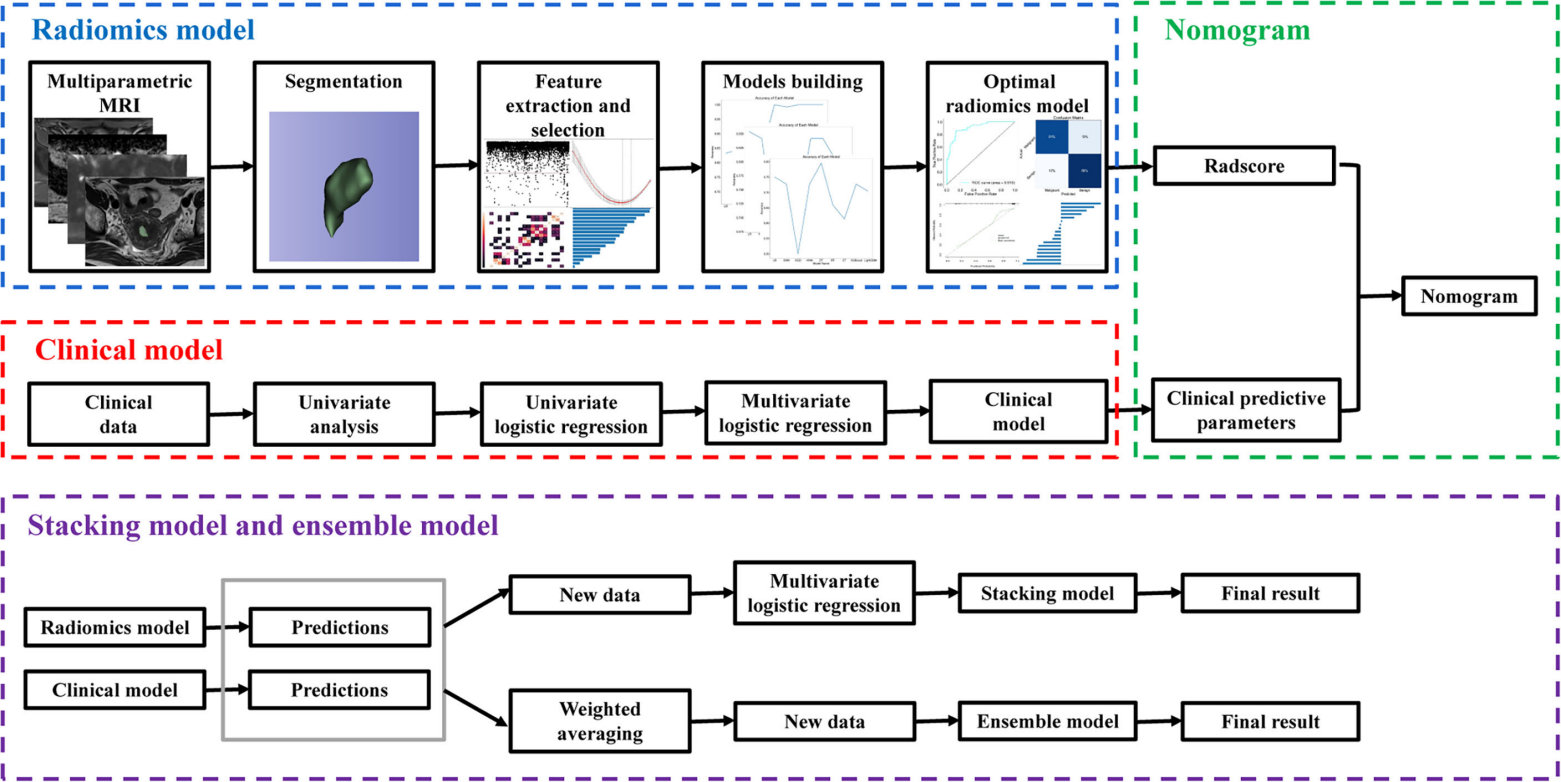
The overall workflow of this study.

Through the nomogram, stacking model, or ensemble model, the clinical and radiomics features were combined, so as to achieve model fusion. All model building was implemented in Python (https://www.python.org/getit/), and the detailed process of model building is shown in **Figure 1**. The AUC, accuracy, sensitivity, specificity, and calibration curve were used as metrics to assess the performance and goodness of fit of the models.

### Clinical application of the models

One radiologist (reader 3, with 30 years of experience in pelvic MRI) who was blind to the clinicopathological information of the patient independently reviewed the MR images to diagnose stage IA EC and benign uterine disease in the training and validation groups. The AUC, accuracy, sensitivity, and specificity of the radiologist were calculated. Clinical decisive curve (CDC), net reclassification index (NRI), and integrated discrimination index (IDI) were performed to estimate the clinical usefulness and net benefit of different models and the radiologist by comparing the actual treatment options of patients.

### Correlations between radiomics features and immunohistochemical findings

In order to explore the correlation between radiomics information and histological characteristics, Spearman correlation coefficients were used to evaluate the correlations between the selected radiomics features and immunohistochemical findings.

### Statistical analysis

All statistical tests were performed using SPSS 26.0 (IBM, New York, USA), R software 4.1.2 (https://www.r-project.org/), and Python 3.9.7 (https://www.python.org/). Continuous variables and categorical variables were respectively expressed as mean value ± standard deviation and counts. The Kolmogorov–Smirnov test was used to check the normality of the continuous data distribution. Continuous variables were analyzed using one-way ANOVA, Mann–Whitney *U* test, or Kruskal–Wallis test. Categorical variables were compared using the Chi-square test or Fisher’s exact test. Univariate and multivariate LR analyses were used to filtrate the clinical predictors and model building. A *p*-value less than 0.05 was considered statistically significant. Pearson correlation analyses were performed to assess correlations between continuous variables, and Spearman correlation analyses were used to evaluate the correlations between continuous variables and ranked data. If *p* < 0.05, there were correlations between the variables.

## Results

### Clinical parameters

A total of 371 patients were divided into the training group (245 patients from center A), the internal validation group (82 patients from center A), and the external validation group (44 patients from centers B). The clinicopathological characteristics of incorporated patients are listed in **Table 2**. The 371 patients included 234 patients with stage IA EC and 137 patients with benign endometrial lesions. Three hundred and twenty patients were treated following the protocol for EC and 51 patients for benign endometrial disease. A total of 112 (30.2%) patients received inappropriate treatment, including 13 (3.5%) patients with stage IA EC who were undertreated and 99 (26.7%) patients with benign endometrial lesions who were overtreated. Univariate analysis showed that the mean age of patients with stage IA EC (51.65 ± 7.94) was significantly older than that of patients with benign uterine lesions (48.12 ± 8.35) in the training group (*p* = 0.001). Compared with benign endometrial lesions, there were more patients with irregular vaginal bleeding and menopause in stage IA EC (*p* < 0.05). No significant differences in metabolic syndrome, BMI, CA125, and CA199 between patients with stage IA EC and benign endometrial lesions were shown (*p* > 0.05). According to the univariate and multivariate LR analysis, age and irregular vaginal bleeding were the valid predictive parameters.

**Table 2:** Clinical and histological characteristics for patients.

	Training group	Internal validation group	External validation group	*p*
Total number	245	82	44	
Patients				
Stage IA endometrial cancer	155	53	26	0.824
Benign endometrial lesions	90	29	18	
Histological subtypes				
Endometrioid adenocarcinoma	155	53	26	0.673
Endometrial hyperplasia	60	21	13	
Endometrial polyp	17	5	5	
Endometrial hyperplasia+polyp	13	3	0	
Age at diagnosis (years)	50.36 ± 8.26	50.52 ± 9.80	54.32 ± 9.34	0.136
Menopause (yes/no)	106/139	33/49	24/20	0.286
Irregular vaginal bleeding (yes/no)	135/110	34/48	36/8	<0.001
Metabolic syndrome (yes/no)	66/179	17/65	13/31	0.468
BMI (kg/m^2^)	25.11 ± 4.38	24.44 ± 3.54	24.80 ± 4.03	0.485
CA125 (U/ml)	37.29 ± 72.77	34.63 ± 64.67	29.52 ± 27.10	0.292
CA199 (U/ml)	23.86 ± 63.44	26.53 ± 46.34	28.39 ± 45.58	0.037

BMI, body mass index.

### Feature selection and optimal machine learning algorithm

**Figure 2:**
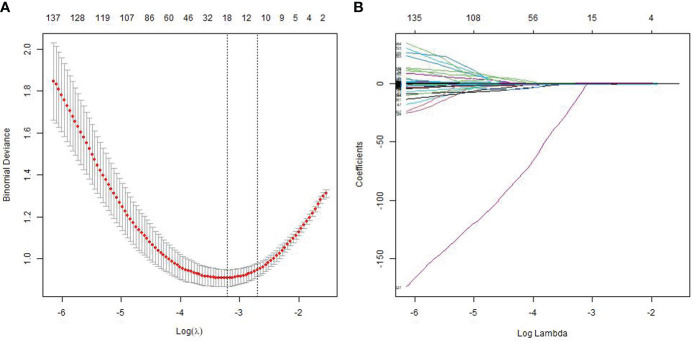
Feature selection using the least absolute shrinkage and selection operator (LASSO) regression model. The cross-validation plot **(A)** and the coefficient profile plot **(B)**.

Among all the extracted features, 3,356 features were excluded because the ICC values between observers or within observers were <0.75. There were 847 features retained after the Pearson correlation analysis. Finally, the LASSO classifier selected 18 features as shown in **Figure 2**.

**Figure 3:**
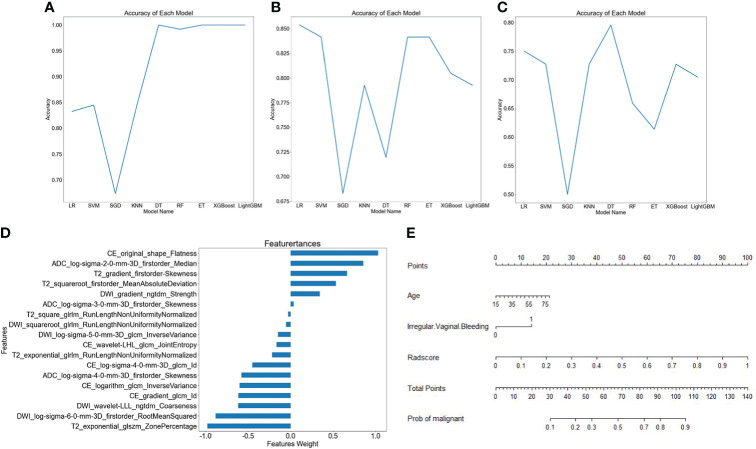
Different model building. Broken line graphs of accuracy for different machine learning algorithms in the training group **(A)**, the internal validation group **(B)**, and the external validation group **(C)**. Bar chart of feature weight for the logistic regression model **(D)**. Nomogram of the training group **(E)**.

The AUC and accuracy of radiomics models constructed by nine machine learning algorithms are shown in **Table 3**, and the broken line graphs of accuracy for different algorithms in the training group, internal validation group, and external validation group are presented in **Figures 3A**–**C**. The LR algorithm showed the highest average AUC (0.854) in the validation groups, and also had the highest average accuracy (0.802). Therefore, LR was considered to be the optimal machine learning algorithm for radiomics model building. The radscore was calculated based on the coefficients and intercepts obtained from the LR model. The selected features and weights are shown in **Figure 3D**. The top four features that contribute most to the radiomics model were CE_original_shape_flatness, T2_exponential_GLSZM_zone percentage, DWI_LOG-sigma-6-0-mm-3D_first order_root mean squared, and ADC_LOG-sigma-2-0-mm-3D_first order_median, respectively.

**Table 3:** The performance of various machine learning algorithms.

	Training group		Internal validation group		External validation group		Validation groups	
	AUC	Accuracy	AUC	Accuracy	AUC	Accuracy	Average AUC	Average Accuracy
LR	0.921	0.832	0.910	0.853	0.798	0.750	0.854	0.802
SVM	0.919	0.844	0.902	0.841	0.796	0.727	0.804	0.784
SGD	0.887	0.804	0.854	0.792	0.705	0.636	0.780	0.714
KNN	0.923	0.844	0.881	0.792	0.757	0.727	0.819	0.760
DT	1	1	0.720	0.719	0.795	0.795	0.758	0.757
RF	1	0.991	0.867	0.841	0.700	0.659	0.784	0.750
ET	1	1	0.905	0.841	0.675	0.613	0.790	0.727
XGBoost	1	1	0.889	0.804	0.813	0.727	0.851	0.766
LightGBM	1	1	0.884	0.792	0.795	0.704	0.840	0.748

AUC, area under the curve; LR, logistic regression; SVM, support vector machine; SGD, stochastic gradient descent; KNN, K nearest neighbor; DT, decision tree; RF, random forest; ET, extremely randomized trees; XGBoost, eXtreme Gradient Boosting; LightGBM, Light Gradient Boosting Machine.

### Performance and clinical application of different models

**Figure 4:**
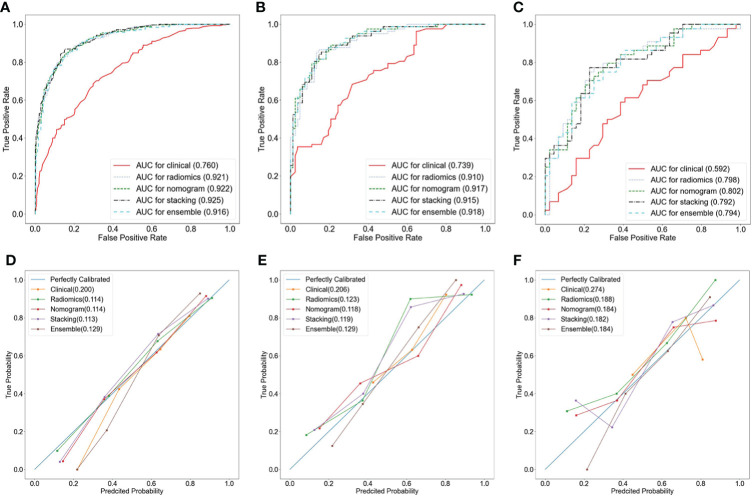
Receiver operator characteristic (ROC) curves **(A–C)** and calibration curves **(D–F)** of different models in the training group **(A, D)**, the internal validation group **(B, E)**, and the external validation group **(C, F)**.

A nomogram was constructed by using the clinical predictive parameters (age and irregular vaginal bleeding) and the radscore (**Figure 3E**). The diagnostic performance of each model and radiologist is displayed in **Table 4**. **Figure 4** shows ROC curves and calibration curves of different models. In the training group, the AUCs of the clinical model, radiomics model, nomogram, stacking model, ensemble model, and radiologist were 0.760, 0.921, 0.922, 0.925, 0.916, and 0.769, respectively. In the internal validation group, they were 0.739, 0.910, 0.917, 0.915, 0.918, and 0.768, respectively. In the external validation group, they were 0.592, 0.798, 0.802, 0.792, 0.794, and 0.628, respectively. According to the calibration curves, the Brier scores of the clinical model, radiomics model, nomogram, stacking model, and ensemble model were 0.200, 0.114, 0.114, 0.113, and 0.129, respectively in the training group. They were 0.206, 0.123, 0.118, 0.119, and 0.129, respectively, in the internal validation group, and they were 0.274, 0.188, 0.184, 0.182, and 0.184, respectively, in the external validation group. The radiomics model, nomogram, stacking model, and ensemble model demonstrated good goodness of fit due to their Brier scores being <0.25.

**Table 4:** Diagnostic efficiency and clinical benefit of different models.

	Models	AUC	Accuracy	Sensitivity	Specificity	NRI (*p*)	IDI (*p*)
Training group	Clinical model	0.760	0.694	0.690	0.700	0.130 (0.082)	0.023 (<0.001)
	Radiomics model	0.921	0.833	0.838	0.833	0.414 (<0.001)	0.393 (<0.001)
	Nomogram	0.922	0.841	0.877	0.800	0.429 (<0.001)	0.396 (<0.001)
	Stacking model	0.925	0.853	0.903	0.800	0.451 (<0.001)	0.498 (<0.001)
	Ensemble model	0.916	0.837	0.832	0.833	0.410 (<0.001)	0.397 (<0.001)
	Radiologist	0.769	0.816	0.948	0.589	0.319 (<0.001)	0.242 (<0.001)
Internal validation group	Clinical model	0.739	0.683	0.528	0.896	−0.163 (0.251)	−0.034 (0.523)
	Radiomics model	0.910	0.854	0.868	0.862	0.307 (0.011)	0.341 (<0.001)
	Nomogram	0.917	0.817	0.887	0.827	0.395 (0.001)	0.362 (<0.001)
	Stacking model	0.915	0.841	0.887	0.828	0.395 (0.001)	0.356 (<0.001)
	Ensemble model	0.918	0.817	0.887	0.828	0.395 (0.001)	0.366 (<0.001)
	Radiologist	0.768	0.780	0.811	0.724	0.216 (0.065)	0.137 (0.018)
External validation group	Clinical model	0.592	0.591	0.500	0.611	−0.068 (0.572)	−0.004 (0.780)
	Radiomics model	0.798	0.750	0.731	0.833	0.423 (0.024)	0.272 (<0.001)
	Nomogram	0.802	0.727	0.731	0.778	0.368 (0.049)	0.234 (0.001)
	Stacking model	0.792	0.773	0.769	0.778	0.423 (0.024)	0.255 (<0.001)
	Ensemble model	0.794	0.705	0.769	0.778	0.368 (0.049)	0.241 (0.001)
	Radiologist	0.628	0.682	0.923	0.333	0.188 (0.220)	0.099 (0.054)

AUC, area under the curve; NRI, net reclassification index; IDI, integrated discrimination index.

**Figure 5:**
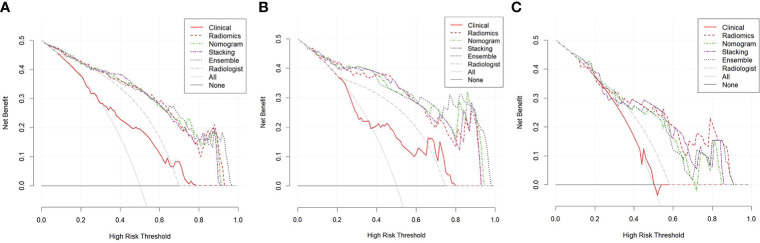
Clinical decision curves (CDCs) of different models and the radiologist in the training group **(A)**, the internal validation group **(B)**, and the external validation group **(C)**.

The CDCs of the different models and the radiologist are presented in **Figure 5**, and the NRI and IDI are shown in **Table 3**. The results showed that the radiomics model, nomogram, stacking model, and ensemble model for predicting stage IA EC added benefit and performed better than the actual treatment options in the training and validation groups (*p* < 0.05). In the training group, the NRI and IDI of the clinical model, radiomics model, nomogram, stacking model, ensemble model, and radiologist were 0.130 and 0.023, 0.414 and 0.393, 0.429 and 0.396, 0.451 and 0.498, 0.410 and 0.397, and 0.319 and 0.242, respectively. In the internal validation group, they were −0.163 and −0.034, 0.307 and 0.341, 0.395 and 0.362, 0.395 and 0.356, 0.395 and 0.366, and 0.216 and 0.137, respectively. In the external validation group, they were −0.068 and −0.004, 0.423 and 0.272, 0.368 and 0.234, 0.423 and 0.255, 0.368 and 0.241, and 0.188 and 0.099, respectively.

### Correlations between radiomics features and immunohistochemical findings

**Figure 6:**
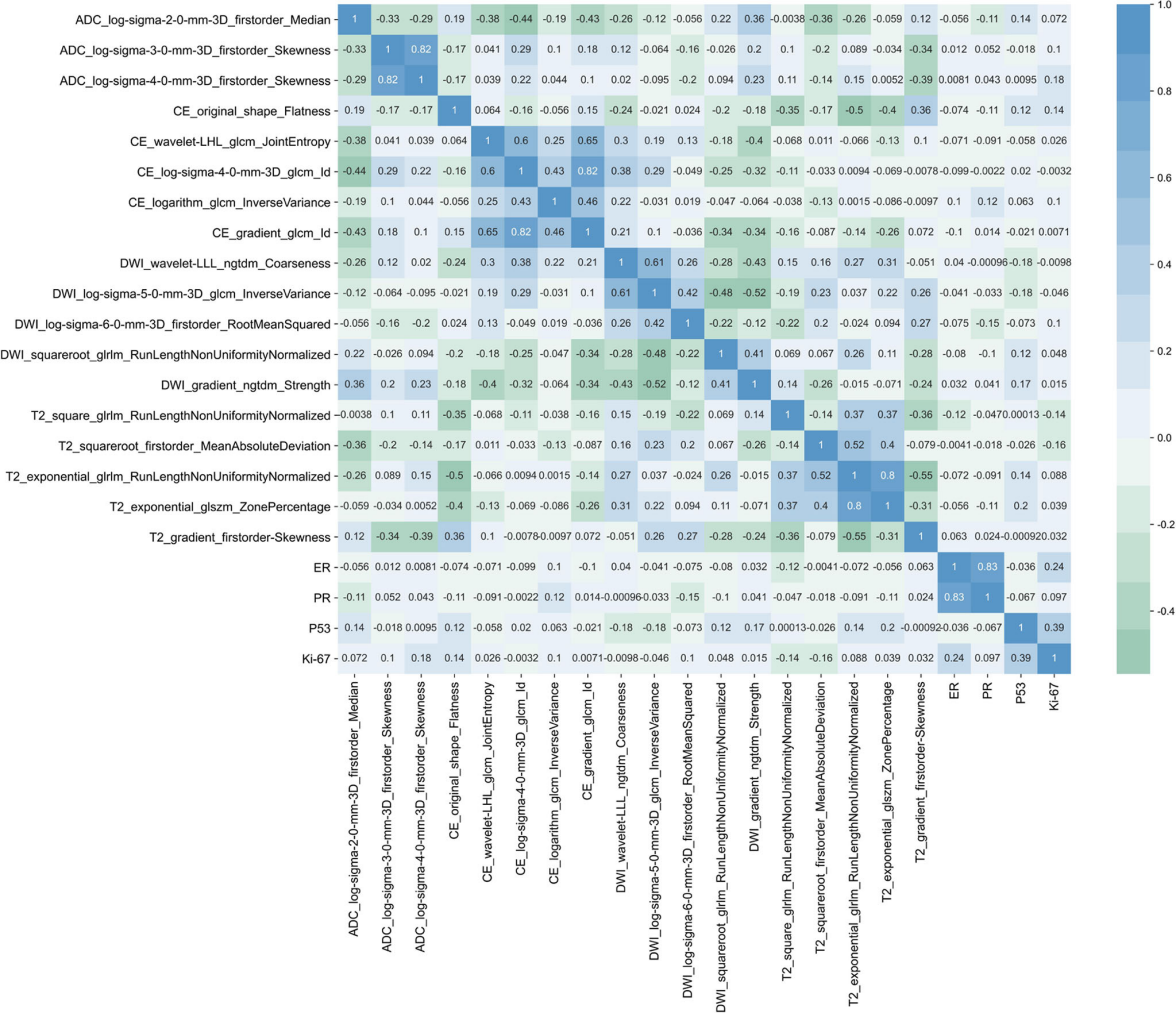
Heatmap of the correlations between the selected radiomics features and ER, PR, P53, and Ki-67.

**Figure 7:**
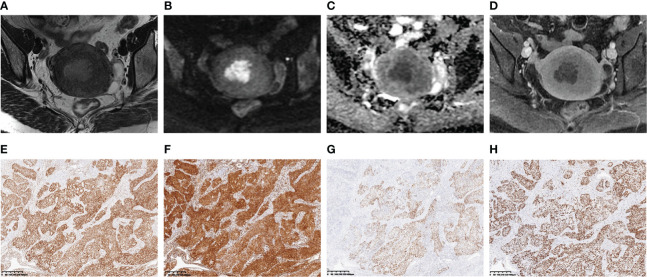
A 46-year-old woman with stage IA endometrial cancer (EC) whose main clinical complication was irregular vaginal bleeding for 3 months. The selected MR images for lesion segmentation included uterus-axial T2-weighted imaging (T2WI) **(A)**, diffusion-weighted imaging (DWI) **(B)**, apparent diffusion coefficient (ADC) map **(C)**, and late contrast-enhanced T1-weighted imaging (LCE-T1WI) **(D)**. The estrogen receptor (ER) **(E)**, progesterone receptor (PR) **(F)**, P53 **(G)**, and Ki-67 **(H)** immunohistochemical staining (40×) showed 90%, 90%, focal, and 80% positive cells, respectively.

A heatmap (**Figure 6**) showed that the selected radiomics features were not correlated with immunohistochemical findings (ER, PR, P53, and Ki-67) (all *p* > 0.05). The selected sequences for lesion segmentation and pathological and immunohistochemical pictures are presented in **Figure 7**.

## Discussion

In the study, age and irregular vaginal bleeding were the valid predictive parameters in the clinical model. On the basis of several common machine learning algorithms, the diverse multiparametric MRI-based radiomics models were developed to differentiate stage IA EC from benign endometrial lesions, and the LR algorithm model was selected as the optimal radiomics model with the highest AUC and accuracy. Compared with the clinical model and the radiologist, the optimal radiomics model and the compositive models combining clinical parameters with radiomics features, like the nomogram, stacking model, and ensemble model, showed better diagnostic performance and achieved good clinical net benefits. The nomogram had a higher AUC than the optimal radiomics model, and revealed more stable discrimination efficiency and better generalization ability than stacking and ensemble models.

The standard surgery of early-stage EC is total hysterectomy with bilateral salpingo-oophorectomy with or without lymphadenectomy/radiotherapy/chemotherapy (30), while the treatment for benign endometrial lesions is a minimally invasive approach compared to hysterectomy, such as hysteroscopic resection or conservative treatment (31, 32). In this study, 3.5% of patients with stage IA EC had undergone inadequate surgery and 26.7% of patients with benign endometrial lesions had undergone overtreatment. As a consequence, the rationalization of treatment options is crucial for patients with stage IA EC and benign mimickers. The most common symptom of EC is irregular vaginal bleeding, which often occurs in the early stage, and the American Cancer Society recommended that all women older than 65 years should be advised to seek risk evaluation of EC if bleeding occurs (33). Therefore, age and irregular vaginal bleeding could be used as effective clinical predictors of stage IA EC. Benign endometrial lesions, such as endometrial hyperplasia and polyps, are highly prevalent in postmenopausal women; symptoms include abnormal uterine bleeding (31, 32). Due to the overlapping clinical features of benign endometrial lesions and EC, the AUC and the diagnostic accuracy of clinical model on the training group and validation groups were low.

In radiomics, the digital medical images that hold information of tumor pathophysiology are transformed into quantitative high-dimensional data to improve medical decision-making, and are gaining importance in cancer research (8). In this study, the radiomics models had high diagnostic performance, which was consistent with the research of Chen et al. (24). The models with high efficiency and reliability are fundamental factors driving the success of radiomics (34), and the recognition of optimal machine learning methods for radiomics models is crucial (35); thus, multiple machine learning algorithms should be employed. We trained nine common classification algorithms, namely, LR, SVM, SGD, KNN, DT, RF, ET, XGBoost, and LightGBM, in model establishment. LR performed best among all classifiers, and the reason might be that complex models required more training samples (36). The optimal radiomics model modeled by LR had higher AUCs and diagnostic accuracies than those of the clinical model and the radiologist in this study. This result further confirmed that radiomics could be a problem-solving tool when there is a dilemma in clinical diagnosis and the observation of conventional imaging (8).

Unlike the study of Chen et al. (24), CE-MRI was included and extracted features in our study. The top four vital features in the optimal radiomics model were from CE-MRI, T2WI, DWI, and the ADC map, respectively. Due to the differences in vascular permeability and microvessel density between EC and benign lesions, most ECs showed early maximal enhancement and late gradual washout, and frequently showed lower signal intensity than the myometrium on LCE-MRI. In contrast, benign lesions showed delayed persistent enhancement pattern, and tended to show higher signal intensity than the myometrium on LCE-MRI (37). The shape of benign and malignant endometrial lesions might be more clearly shown on LCE-MRI. Flatness shows the relationship between the largest and smallest principal components in the ROI shape (8). In consequence, CE_original_shape_flatness was the most contributing feature. Endometrial polyp and hyperplasia are rich in fibrous stromal structures and endometrial glands (1). Specific MRI findings such as a fibrous tissue (hypointensity on T2WI) and intratumoral cysts (hyperintensity on T2WI) might be useful to differentiate benign endometrial lesions from EC (1). Additionally, the zone percentage of GLSZM features represents the coarseness of the texture, and can better reflect the heterogeneity of different tumors (8). Thus T2_exponential_GLSZM_zone percentage was also an important feature. A previous study had suggested that DWI with ADC values were a potential quantitative and qualitative tool for differentiating between early-stage EC and benign mimickers (38). On DWI, benign endometrial lesions showed low signal intensity, which was an important point in differentiating them from EC that showed high signal intensity due to relatively high cellularity (39). Nevertheless, the top two important features were not derived from DWI and the ADC map in this study. The possible reason was that DWI was acquired in different scanners, which might lead to inconsistency in image quality and ADC estimation across vendors, although the models remained effective after cross-validation in datasets from scanners with different manufacturers or with different Tesla. Another possible reason was that benign uterine lesions rich in cystic areas and mucus might increase DWI signal intensity due to the influence of the T2-penetration effect, and hemorrhagic areas and mucous components could reduce the signal intensity of the ADC map, which would lead to a slight difference between DWI and ADC maps of benign and malignant endometrial lesions, thus resulting in the reduction of the weight of their features.

Gatenby et al. (40) believed that radiomics features could offer information on the phenotype and microenvironment of tumors, which was complementary to other data like clinical parameters. Radiomics features combined with clinical parameters and other pertinent data can produce accurate robust evidence-based clinical-decision support systems (8). In this study, according to ROCs, CDCs, NRI, and IDI, the compositive models modeled by clinical parameters and radiomics features, such as the nomogram, stacking, and ensemble models, showed better diagnostic performance and achieved better clinical net benefits than the clinical model and the radiologist. Compared with the radiomics model, the nomogram had a higher AUC. Yan et al. (11) developed an MRI- and clinical-based radiomics nomogram to preoperatively assess high-risk EC, and obtained a similar result to this study, which was the prediction efficiency of nomogram was better than that of the radiomics model. The advantage of the ensemble strategy was that it can reduce the variance and bias of the model by a powerful process of majority vote or group averaging, and it improves the robustness and generalizability of the model in prediction and classification (27). A recent study had confirmed that the two-tier stacking model could further improve the generalization ability of the radiomics model compared with the single model (41). In the present study, the diagnostic performance of the stacking model and ensemble model was similar with that of the nomogram and better than that of the radiomics model in the internal validation group, whereas the AUCs of the stacking model and ensemble model were lower than those of the nomogram and radiomics model in the external validation group. Therefore, the nomogram presented more excellent and stable differential diagnostic efficiency than stacking and ensemble models with good reproducibility and reliability.

There were some limitations in the study. First, this study only collected patients from two centers. Patients from more centers need to be included to improve the universality of the model in clinical application. Second, the MRI systems and scanning parameters were not uniform, and it may influence the models’ results, especially in the external validation group. Third, only traditional radiomics features were extracted; the deep-learning-based features were not investigated. In the future, we will conduct in-depth learning combined with traditional radiomics to build models. Last, manual lesion segmentation is time-consuming and is easily affected by the experience of readers; automatic or semiautomatic methods that delineate lesions more accurately need to be explored in the future.

## Conclusions

The multiparametric MRI-based radiomics models can be conveniently used for preoperative identification of patients with stage IA EC and benign endometrial lesions, and the model established by the LR algorithm has the highest accuracy. Incorporating radiomics and clinical parameters (age and irregular vaginal bleeding) into a combined model to estimate patients was more accurate than the clinical model and the radiologist. This study is beneficial in noninvasively identifying benign and malignant endometrial lesions that are difficult to determine by clinicians and radiologists before surgery, avoiding misdiagnosis and missed diagnosis, and providing a basis for the patient protocol of individualized diagnosis and treatment.

## Data availability statement

The raw data supporting the conclusions of this article will be made available by the authors, without undue reservation.

## Ethics statement

The studies involving human participants were reviewed and approved by Medical Ethics review committee of the First People’s Hospital of Yunnan Province. Written informed consent for participation was not required for this study in accordance with the national legislation and the institutional requirements.

## Author contributions

QB designed the study, performed the statistical analysis, and wrote the manuscript. YXW collected the data and performed the statistical analysis. YD, YL, and YP collected the data. YS and YZW revised the manuscript. KW guaranteed the integrity of the entire study. All authors approved the submitted version of the manuscript.

## Acknowledgments

We sincerely thank Programmer Guanghong Yang and Platform Onekey AI for Code consultation of the study.

## Conflict of interest

Authors YS and YW were employed by Siemens Healthineers.

The remaining authors declare that the research was conducted in the absence of any commercial or financial relationships that could be construed as a potential conflict of interest.

## Publisher’s note

All claims expressed in this article are solely those of the authors and do not necessarily represent those of their affiliated organizations, or those of the publisher, the editors and the reviewers. Any product that may be evaluated in this article, or claim that may be made by its manufacturer, is not guaranteed or endorsed by the publisher.
